# The Helping Alliance Questionnaire for Children and Their Caregivers—Validation in a German Sample of Pediatric Chronic Pain Patients

**DOI:** 10.1002/jclp.70152

**Published:** 2026-05-07

**Authors:** Lisa‐Marie Rau, Amelie Florentine Schmidt, Julia Wager

**Affiliations:** ^1^ Department of Children's Pain Therapy and Paediatric Palliative Care, School of Medicine, Faculty of Health Witten/Herdecke University Witten Germany; ^2^ German Paediatric Pain Centre Children's and Adolescents' Hospital Datteln Datteln Germany; ^3^ PedScience Research Institute Datteln Germany

**Keywords:** chronic pain, health personnel, parents, pediatrics, therapeutic alliance

## Abstract

**Objectives:**

The “helping alliance” between patients, caregivers, and healthcare professionals (HCPs) plays an important role in the evaluation of therapeutic interventions. The aim of this study was to develop a German version of the Helping Alliance Questionnaire for pediatric patients (HAQ‐P) and caregivers (HAQ‐CG).

**Methods:**

The questionnaire was adapted from the original version of the HAQ for adults and improved using feedback from HCPs, patients with chronic pain and their caregivers. It was then validated in a sample of *N* = 216 patients with chronic pain aged 8–17 years, along with their caregivers and HCPs.

**Results:**

Confirmatory factor analysis revealed a good model fit for the 2‐factor structure of the HAQ‐P and HAQ‐CG, with the two subscales *relationship* and *outcome satisfaction* demonstrating good to excellent internal consistency across all subgroups. Multi‐group analyses showed that the model structure was similar for patients, caregivers, and HCPs. However, means of the three groups differed systematically (e.g., caregivers reported higher satisfaction), thus not allowing for direct comparison of group means.

**Conclusions:**

The HAQ‐P and HAQ‐CG have strong psychometric properties, and may be useful in clinical practice and research for monitoring, predicting, and improving therapeutic outcomes.

## 1 Introduction

The relationship between patient and therapist is crucial for the success of therapeutic interventions (Flückiger et al. 2015). When therapists build a strong alliance with pediatric patients and their caregivers, treatment outcomes are generally better. This includes improved pain characteristics, higher treatment satisfaction, and lower dropout rates (Accurso et al. 2013; Burns et al. 2015). The Helping Alliance Questionnaire (HAQ; Luborsky 1984) is an established tool for measuring this therapeutic relationship. In contrast to other measures such as the California Psychotherapy Alliance Scale (CALPAS; Gaston 1991) or the Vanderbilt Psychotherapy Process Scale (VPPS; Strauß et al. 1992), the HAQ encompasses the concept of satisfaction with treatment. In the context of pediatric chronic pain research, satisfaction with treatment is usually measured with the single item on treatment satisfaction of the German Pediatric Pain Questionnaire (Schroeder et al. 2010), which previous studies have found ceiling effects for (Stahlschmidt et al. 2018). Consisting of two scales, the HAQ assesses both the relationship between the therapist and patient and satisfaction with the therapeutic outcome, offering the advantage of overcoming the challenge of measuring treatment satisfaction.

Bassler et al. (1995) developed a German version of the HAQ for adult patients and their healthcare professionals (HCPs), applicable in both inpatient and outpatient settings (Nübling et al. 2017). While there is a French version of the HAQ for children (Kermarrec et al. 2006) and an English version for measuring the alliance between caregivers and HCPs (Accurso et al. 2013), no such measures exist in German. Therefore, the aims of the present study were to develop a German version of the HAQ for both the patient‐HCP alliance (HAQ‐P) and the caregiver‐HCP alliance (HAQ‐CG) and to validate it in a sample of patients with chronic pain.

Pain conditions are defined as chronic if they persist or recur for at least 3 months or longer than expected for normal healing (Harvey 1995; Treede et al. 2019). Chronic pain among youths is usually primary, meaning there is no physical tissue damage that sufficiently explains the pain (Alp et al. 2010; Thornton et al. 2016). Chronic pain poses a significant health issue, affecting 20%–30% of children and adolescents. Between 6% and 8% are highly impacted by their pain, that is, they miss out on daily activities such as going to school or experience emotional impairment such as anxiety or depression (Grothus et al. 2024; Könning et al. 2021). The state of the art method for treating chronic pain is a multimodal pain treatment that includes medical and psychosocial elements (Claus et al. 2022; Stahlschmidt et al. 2016). A key part of this treatment is specialized pain psychotherapy that involves caregivers, as they significantly influence the child's pain condition (Simons et al. 2008; Stahlschmidt et al. 2016). Measuring the helping alliance may improve treatment outcomes and their predictability, providing HCPs with a tool to monitor and improve their therapeutic alliance with patients and their caregivers.

In the current study, it was hypothesized that adapting the HAQ for the pediatric setting would replicate the two‐factor structure of the original HAQ (*relationship* and *outcome satisfaction*) and show good psychometric properties (factorial validity). A moderate correlation between HAQ‐P and HAQ‐CG scores for patients, caregivers, and healthcare professionals was expected. Additionally, it was hypothesized that HAQ‐P and HAQ‐CG scores would correlate positively with related constructs, such as overall treatment satisfaction, patient pain self‐efficacy, and parental pain‐related behavior and confidence. A negative correlation between HAQ‐P scores and pain characteristics was expected (convergent validity).

## 2 Methods

The present study consists of a two‐step process. First, a pediatric German version of the HAQ was developed and tested. Second, the adapted questionnaire was validated in a sample of *N* = 216 pediatric patients with chronic pain, their caregivers, and healthcare professionals.

### 2.1 Phase 1: Questionnaire Development

To develop the questionnaire, pediatric chronic pain researchers and healthcare professionals adapted the adult German HAQ as well as the original version by Bassler et al. (1995). This process resulted in four sets of items: Patients rating the patient‐HCP alliance, HCPs rating the patient‐HCP alliance (HAQ‐P), caregivers rating the caregiver‐HCP alliance, and HCPs rating the caregiver‐HCP alliance (HAQ‐CG).

The questionnaires were then tested with *N* = 4 patients with chronic pain and *N* = 4 caregivers in cognitive interviews. Cognitive interviewing improves content validity by having the target groups read and answer the items (Crombez et al. 2023). Participants are asked to think aloud and comment on their understanding and any challenges encountered while completing the questionnaire. Interviews with patients were conducted with two 9‐year‐old girls, a 10‐year‐old girl and a 17‐year‐old boy. The participating caregivers were three mothers aged 41, 48 and 50 years as well as a father aged 42 years. Within both groups, participants gave similar feedback, that is, they found the same words and sentences difficult to understand. As a result of the interviews, as per the patients’ and caregivers’ suggestions, identified words and phrases were replaced, simplified or specified.

Given that the HAQ's original version has been validated in HCPs, no difficulties in comprehensibility from their perspective could be assumed. Instead, HCPs were asked on their expert opinion of the fit of the adapted questionnaire to the pediatric setting. Their feedback was consulted to ensure face and content validity of questionnaires from practitioners’ perspective.

The final versions of the questionnaires adapted from Bassler et al. (1995), along with non‐validated English translations, are available in Tables S1 and S2.

### 2.2 Phase 2: Validation

#### 2.2.1 Sample

**Figure 1:**
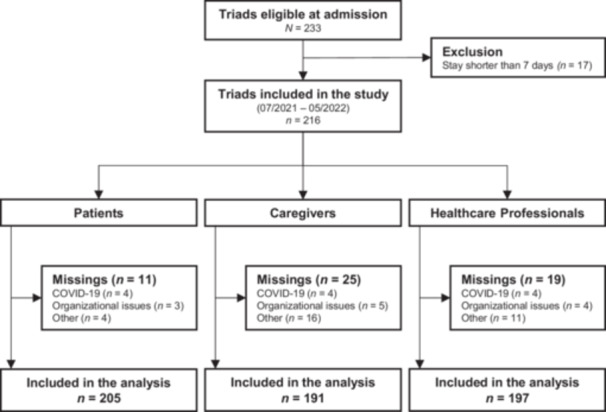
*P*articipants included in analyses. *Note:* Missing values due to COVID‐19 indicate that participants left the inpatient setting due to a positive PCR‐test.

Data for the validation were collected from patients with chronic pain aged 8 to 17 years as well as their caregivers and HCPs after discharge from inpatient intensive interdisciplinary pain treatment (IIPT) with a minimum stay of 7 days at a children's hospital in Germany. HCPs filled out the questionnaire for several of their patients. Additional clinical information regarding patient's pain characteristics were assessed at admission to the IIPT. To ensure stable results in confirmatory factor analyses, approximately 200 datasets were needed (Kline 2023; Kyriazos 2018). To achieve this sample size, all patients admitted to treatment between July 2021 and May 2022 were screened for eligibility. Patients were excluded if their inpatient stay was shorter than 7 days, resulting in a sample of *N* = 216 families. Of these, 205 patient, 191 caregiver, and 197 HCP questionnaires were completed. A detailed overview of the available data are provided in Figure 1.

Of the 205 pediatric patients, 150 (73.2%) were female, 52 (25.4%) male, and 3 (1.5%) diverse. Their mean age was 14.42 years (*SD* = 1.98), ranging from 9 to 17 years. Among the 191 caregivers, 122 (63.9%) identified as female, 68 (35.6%) as male, and 1 (0.5%) as diverse. The patients' mean length of stay was 21.08 days (*SD* = 4.87), ranging from 7 to 38 days. Data of *n* = 192 patients were collected at admission, missing data of *n* = 13 patients were due to organizational issues. Pain characteristics reported by patients can be found in Table 1.

**Table 1:** Pain characteristics of patients.

Pain characteristics	Patients' responses (*N* = 192)
**Pain onset**	
More than 2 years ago	*n* = 96 (50%)
1–2 years ago	*n* = 43 (22%)
6–12 months ago	*n* = 27 (14%)
3–6 months ago	*n* = 21 (11%)
Less than 3 months ago	*n* = 5 (3%)
**Pain frequency**	
Continuous *	*n* = 88 (46%)
Daily	*n* = 51 (27%)
Several times a week	*n* = 37 (19%)
Once a week	*n* = 9 (5%)
Once a month	*n* = 5 (3%)
Once in the last 3 months	*n* = 2 (1%)
**Number of main pain locations**	
One pain location	*n* = 160 (83%)
Two ore more pain locations	*n* = 32 (17%)
**Main pain location**	
Head	*n* = 112 (58%)
Abdomen	*n* = 39 (20%)
Chest	*n* = 8 (4%)
Musculoskeletal	*n* = 74 (39%)
**Mean pain intensity**	*M* = 6.32 (*SD* = 1.75; 0‐10)
**Maximum pain intensity**	*M* = 8.34 (*SD* = 1.46; 0‐10)

*Note:* Patients were able to select multiple pain locations. Mean and maximum pain intensity refers to the pain experienced within the last four weeks.

* Continuous means that patients experience their pain as always being present and never stopping; in contrast to ‘daily,’ where patients could for example experience pain for two hours each day.

The caregivers’ mean age was 47.16 years (SD = 5.41), ranging from 33 to 68 years. No data of HCPs were collected to ensure their anonymity. It was important for HCPs to know that the aim of the study was to evaluate the questionnaire and not them personally.

#### 2.2.2 Instruments

The study included surveys for patients, caregivers, and HCPs. All surveys included the HAQ‐P or HAQ‐CG and an assessment of pain severity. Additionally, patients and caregivers completed the following instruments: both groups evaluated global treatment satisfaction, patients reported pain self‐efficacy, and caregivers evaluated pain‐related parental behavior and confidence in managing their child's chronic pain. All variables were measured via self‐reports.

##### 2.2.2.1 Demographic Variables

Both patients and caregivers reported their age and gender (girl/female, boy/male, diverse).

##### 2.2.2.2 Pain Characteristics

At admission, pain characteristics of patients were assessed with questions stemming from the German Pediatric Pain Questionnaire (Schroeder et al. 2010). Patients were asked to report when their pain condition began and how often they experience pain. To assess their pain locations, patients reported all body regions they experienced their main pain in (head, abdomen, chest, musculoskeletal). Mean and maximum pain intensity experienced within the last 4 weeks were assessed using a numeric rating scale (NRS) ranging from 0 = *no pain* to 10 = *strongest pain*.

##### 2.2.2.3 HAQ‐P and HAQ‐CG

The German versions of the adapted Helping Alliance Questionnaire for pediatric patients (HAQ‐P) and caregivers (HAQ‐CG) consist of separate questionnaires for each relevant group in this setting (patients, caregivers, HCPs). As shown in Tables S1 and S2, patients and caregivers rate the helping alliance toward HCPs from their own perspective. For instance, caregiver *relationship* questions focus on the relationship between the caregiver and the HCPs, not the child and HCPs. Participants are instructed to rate questions regarding the whole treatment team (doctors, therapists, nurses, etc.), instead of evaluating only one person, to reflect the interdisciplinary aspect of IIPT. Similarly, HCPs rate the helping alliance towards patients and caregivers separately. In this study, HCP questionnaires were completed by the therapists or doctors in charge for the patient.

Each questionnaire consists of 11 items and one global item. The 11 items are answered on a 6‐point scale ranging from 1 (*strongly disagree*) to 6 (*strongly agree*). Six of these items assess the *relationship* between the patient/caregiver and HCP (items 1, 6, 7, 8, 9, 10), while the remaining five items assess *outcome satisfaction* (items 2, 3, 4, 5, 11). The additional global item gauges the perceived change in treatment success since the beginning of the treatment, rated on a 7‐point scale ranging from 1 (*much worse*) to 7 (*much better*) (midpoint: 4—*no change*).

In the current literature, there is inconsistency in how the HAQ score is calculated. Some researchers compute one global score across all 11 items (see e.g., Lutz et al. 2015; Meyer et al. 2015), while others calculate scores for each subscale (see e.g., Michel et al. 2004; Puschner et al. 2005). Although both methods show high internal consistency, previous factor analyses suggest that the two‐factor solution should be preferred (Bassler and Nübling 2015; Bassler et al. 1995). In this study, both approaches were compared in a confirmatory factor analysis. The global item is not part of the HAQ subscales and was therefore used as an external variable to assess validity.

##### 2.2.2.4 Global Treatment Satisfaction

Patients and caregivers rated their overall satisfaction with the treatment using a global item from the German Pain Questionnaire for Children and Adolescents (DSF‐KJ; Schroeder et al. 2010; Stahlschmidt et al. 2018). They answered on an NRS ranging from 0 (*very unsatisfied*) to 10 (*very satisfied*).

##### 2.2.2.5 Chronic Pain Severity

To assess the severity of chronic pain, patients completed three items originally developed as ICD‐11 specifiers by Treede et al. (2019) that were adapted and validated for use in pediatrics by Rau et al. (2025). These items portray the biopsychosocial dimensions of chronic pain according to the ICD‐11 (World Health Organization 2021). These three items assess the (1) intensity, (2) emotional distress, and (3) functional disability of pain experienced in the last 7 days (Rau et al. 2025). Patients rated each item on an NRS from 0 (*no pain*/*not at all*) to 10 (*strongest pain*/*very much*). These three items represent distinct constructs and are therefore not combined into a total score (Rau et al. 2025).

##### 2.2.2.6 Scale for Pain Self‐Efficacy

Pain self‐efficacy was measured using the Scale for Pain Self‐Efficacy (SPaSE; Stahlschmidt et al. 2023). The SPaSE comprises 11 items rated on a 5‐point scale from 0 (*does not apply*) to 4 (*does apply*). In the present sample, the SPaSE demonstrated good internal consistency, with a Cronbach's alpha of 0.92.

##### 2.2.2.7 Pain‐Related Parental Behavior

Pain‐related parental behavior was measured using the inventory for pain‐related parental behavior (ISEV‐E; Hermann et al. 2008). This questionnaire consists of 17 items across three subscales: (1) discouraging behavior (e.g., ‘*When my child is in pain, I become inpatient*.’), (2) solicitous behavior (e.g., ‘*When my child is in pain, I try to encourage them to take a break*.’) and (3) distracting behavior (e.g., ‘*When my child is in pain, I turn on the TV to distract them from the pain*.’). Caregivers rated how often they display these different types of behavior using a 5‐point scale from 1 (*never*) to 5 (*very often*). In the present sample, Cronbach's alpha was 0.77 for the 7‐item *discouraging behavior* subscale, 0.81 for the 6‐item *solicitous behavior* subscale and 0.58 for the 4‐item *distracting behavior* subscale.

##### 2.2.2.8 Global Confidence in Handling Pain

As an additional measure to assess the caregivers’ perspective, a global item regarding confidence in handling the child's pain was implemented in the caregiver questionnaire (‘*How confident are you in handling your child's pain?*’). Responses were recorded on an NRS ranging from 0 (*not confident at all*) to 10 (*very confident*).

#### 2.2.3 Data Analysis

All statistical analyses were conducted using R version 4.2.2 (R Core Team 2024) and RStudio (Posit team 2024). Confirmatory factor analysis was performed using the *lavaan* package in R (Rosseel 2012). The significance level was set to *α* < 0.05 and Bonferroni‐Holm corrections were applied to mitigate errors from multiple testing (Holm 1979).

##### 2.2.3.1 Item and Scale Properties

For the HAQ‐P and HAQ‐CG, item properties such as means, standard deviations, and ranges were calculated for all items. To assess reliability, internal consistency (Cronbach's *α*) was computed for the two subscales *outcome satisfaction* and *relationship*. Scale means, standard deviations, and ranges were determined for all groups and genders.

##### 2.2.3.2 Construct Validity

The questionnaires were tested for construct validity through factorial and convergent validity analyses.

##### 2.2.3.3 Factorial Validity

After confirming statistical assumptions, a confirmatory factor analysis (CFA) was conducted on the HAQ‐P data from patients. Aligning with previous research, a one‐factor model was compared to a two‐factor model (Bassler and Nübling 2015; Lutz et al. 2015; Puschner et al. 2005). Due to a low factor loading of item 11, an additional third model of a two‐factor solution excluding this item was compared to the original model. The criteria for an acceptable model fit were defined as follows: comparative fit index (CFI) and Tucker Lewis Index (TLI) 0.95 or higher, root mean square error of approximation (RMSEA) 0.06 or lower, and standardized root mean square residual (SRMSR) at 0.08 or lower (Hu and Bentler 1999). All CFA calculations utilized a Satorra‐Bentler correction to ensure robust results in non‐normally distributed data (Satorra and Bentler 2010).

The final patient model was first tested across patients’ age groups split by median with a multi‐group factor analysis. This was an iterative process in which the initial model (configural model) was compared to a model with fixed factor loadings between groups (metric model), and then to a model with fixed intercepts between groups (scalar model). Each model was compared to the previous, less restrictive model, with the criterion that a difference in CFI of 0.01 or smaller indicated acceptable restriction. This approach ensures that the model works comparably between groups in terms of factor loadings or intercepts respectively (Hirschfeld and Brachel 2019; Vandenberg and Lance 2000). The same approach was then applied to the HAQ‐P and HAQ‐CG data from all four sources (patients, HCPs regarding patients, caregivers, and HCPs regarding caregivers), to test whether the questionnaires are applicable in all target groups.

##### 2.2.3.4 Convergent Validity

Correlations between HAQ‐P and HAQ‐CG data and the other constructs assessed in this study were calculated to ensure convergent validity. Because the data were not normally distributed, Kendall's tau (*τ*) was used as a more robust tool to calculate correlations. When interpreting Kendall's *τ* values, correlations ranging between |τ | = 0.1 and |τ | = 0.3 are considered small, those between |τ | = 0.3 and |τ | = 0.5 are considered moderate, and correlations of |τ | = 0.5 or higher are considered large (Cohen 1988).

## 3 Results

### 3.1 Item and Scale Properties

In the patient population, the means of items 1 to 11 ranged from *M* = 4.48 (item 10; *similar ideas*) to *M* = 5.30 (item 8; *achieve goals*). Among caregivers, the lowest mean was for item 2 (*M* = 4.71; *treatment helps*), while the highest mean was for item 8 (*M* = 5.55; *achieve goals*). HCPs rated item 11 (*independence*) the lowest, both for patients (*M* = 4.01) and caregivers (*M* = 3.82). The item with the highest mean values for HCPs was item 6 (*dependable HCP*) for both patient evaluation (*M* = 5.28) and caregiver evaluation (*M* = 5.05). Since the global item has a different range than the other 11 items, item properties are not directly comparable. See Table 2 for properties of all items.

**Table 2:** Item‐level HAQ‐P and HAQ‐CG ratings for patients, caregivers, and healthcare professionals.

Item	Patients (*n* = 205)			Caregivers (*n* = 191)			HCP‐P (*n* = 197)			HCP‐CG (*n* = 197)		
	*M*	*SD*	Range	*M*	*SD*	Range	*M*	*SD*	Range	*M*	*SD*	Range
1	5.11	0.98	1–6	5.22	0.83	2–6	4.87	0.84	2–6	4.52	0.91	2–6
2	4.95	1.01	1–6	4.71	1.01	1–6	4.86	0.85	2–6	4.46	0.92	2–6
3	5.08	0.97	1–6	4.99	0.95	1–6	4.90	0.84	2–6	4.58	0.88	1–6
4	4.70	1.13	1–6	5.03	1.00	1–6	4.63	0.82	2–6	4.13	0.99	1–6
5	4.72	1.05	1–6	5.04	0.89	1–6	4.16	0.92	1–6	3.89	0.91	1–6
6	5.08	1.00	1–6	5.16	0.86	2–6	5.28	0.78	4–6	5.05	0.78	3–6
7	4.78	1.12	1–6	5.04	0.96	2–6	4.94	0.75	3–6	4.46	0.93	1–6
8	5.30	0.95	1–6	5.55	0.67	2–6	5.26	0.73	4–6	4.96	0.75	3–6
9	5.02	1.03	1–6	5.13	0.95	1–6	4.68	0.86	2–6	4.34	0.97	1–6
10	4.48	1.19	1–6	4.95	0.98	1–6	4.40	0.90	1–6	4.18	1.01	1–6
11	4.64	1.09	1–6	4.79	0.98	1–6	4.01	1.01	1–6	3.82	0.99	1–6
Global	5.53	1.06	1–7	5.46	1.19	1–6	5.23	0.70	3–7	4.88	0.70	3–6

Abbreviations: HCP‐CG, ratings of healthcare professionals towards caregivers; HCP‐P, ratings of healthcare professionals towards patients; *M*, mean; *SD*, standard deviation.

**Figure 2:**
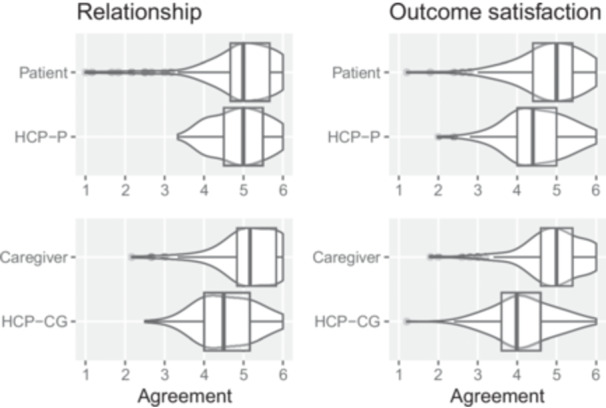
Agreement between ratings of patients, caregivers, and HCP for the HAQ‐P and HAQ‐CG subscales “relationship” and “outcome satisfaction.” Abbreviations: HCP‐CG, healthcare professional rating of helping alliance with caregiver; HCP‐P, healthcare professional rating of helping alliance with patient.

According to Gliem and Gliem (2003), the internal consistency of the *relationship* subscale was excellent for all groups, ranging from *α* = 0.90 in HCPs to *α* = 0.92 in patients. For the *outcome satisfaction* subscale, Cronbach's alpha ranged from *α* = 0.85 in patients and caregivers to *α* = 0.91 in HCPs, indicating good to excellent internal consistency. More details are available in Table 3, which presents Cronbach's alpha, means, and standard deviations for both scales across all groups and genders. Agreement between patient and HCP ratings, as well as between caregivers and HCP ratings, are presented in Figure 2.

**Table 3:** Cronbach's alpha and descriptive results of the two HAQ‐P and HAQ‐CG‐subscales for all subgroups, stratified by gender.

Group	*N*	Relationship				Outcome satisfaction			
		α	*M*	*SD*	*Range*	α	*M*	*SD*	*Range*
Patients	205	0.92	4.96	0.89	1.00–6.00	0.85	4.82	0.84	1.20–6.00
Female	150	0.93	4.93	0.89	1.00–6.00	0.84	4.75	0.84	1.20–6.00
Male	52	0.92	5.05	0.91	2.17–6.00	0.89	4.98	0.81	3.00–6.00
Diverse	3	—	5.33	0.44	5.00–5.83	—	5.40	0.20	5.20–5.60
Caregivers	191	0.91	5.17	0.74	2.17–6.00	0.85	4.91	0.77	1.80–6.00
Female	122	0.92	5.18	0.75	2.17–6.00	0.88	4.94	0.82	1.80–6.00
Male	68	0.90	5.15	0.72	2.67–6.00	0.80	4.86	0.67	3.00–6.00
Diverse	1	—	5.67	—	—	—	5.20	—	—
HCP‐P	197	0.90	4.90	0.66	3.33–6.00	0.91	4.51	0.76	2.00–6.00
HCP‐CG	197	0.90	4.58	0.73	2.50–6.00	0.90	4.18	0.79	1.20–6.00

*Note:* Possible scale range was 1–6. Cronbach's alpha was not calculated in diverse subgroups due to lack of variance.

Abbreviations: α, Cronbach's alpha; HCP‐P, healthcare professional ratings towards their helping alliance with the patients; HCP‐CG, healthcare professional ratings towards their helping alliance with the caregivers; *M*, mean; *SD*, standard deviation.

### 3.2 Construct Validity

#### 3.2.1 Confirmatory Factor Analysis

When comparing the one‐factor model with the two‐factor model using patient data, the model comparison yielded significant results, *X* ^2^_diff_(1, *N* = 205) = 31.45, *p* < 0.001. However, all fit indices strongly indicate a preference for the two‐factor model, as their values lie closer to the predefined thresholds (Hu and Bentler 1999). Detailed fit indices for the compared models are provided in Table 4.

**Table 4:** Model fit per confirmatory factor analysis of the patient sample.

	X^2^	*df*	*p*	CFI	TLI	RMSEA	SRMR
1‐Factor model	188.25	44	< 0.001	0.85	0.81	0.12	0.16
2‐Factor model	111.72	43	< 0.001	0.93	0.91	0.11	0.07
2‐Factor model without item 11	76.91	34	< 0.001	0.95	0.94	0.10	0.05

*Note:* Robust values were computed using Satorra‐Bentler correction. After considering both statistical results and theoretical considerations, the two‐factor model (includes all items) was optimal.

Abbreviations: CFI, Comparative Fit Index; RMSEA, Root Mean Square Error of Approximation; SRMR, Standardized Root Mean Square Residual; TLI, Tucker–Lewis Index.

The factor loadings of the two‐factor model are presented in Table S3 to Table S6. Item 11 (*independence*) had a factor loading of *ʎ* = 0.53, considerably lower than the other items (ranging from *ʎ* = 0.68 to *ʎ* = 0.89). An additional model mimicking the two‐factor model but excluding item 11 was tested against the full two‐factor model. However, despite slightly better fit indices (see Table 4), the comparison between the nested models was significant, X^2^_diff_(9, *N* = 205) = 33.80, *p* < 0.001. This indicates that the model with higher complexity (i.e., the model including item 11) should be preferred. While only one of the four fit indices met the established criteria in the two‐factor structure, both the factor loadings and the excellent internal consistency within the patient sample suggested a good fit of the model. Goretzko et al. (2024) found that it is common for models not to meet strict fit criteria and argue that indices should be used as an orientation rather than as strict guidelines. Additionally, it is important to include theoretical considerations into the choice of a model (Brown 2015). In the case of the HAQ, including the item 11 allows for easier comparison with data of other version of the HAQ. With these considerations taken together, the two‐factor model including item 11 was used for further analyses.

#### 3.2.2 Multi‐Group CFA

To test whether the two factor structure of the HAQ‐P is valid independent of the patients’ age, the patient sample was split by the median age of 15 years, resulting in two age groups: ages 8–14 (*n* = 98) and ages 15–17 (*n* = 107). Results of the multi‐group CFA of age groups are shown in Table 5. The difference in CFI is −0.004 for the metric model and 0.011 for the configural model. According to the threshold established by Vandenberg and Lance (2000), both of these models are acceptable, that is, both the factor structure and intercepts are comparable across age groups.

**Table 5:** Multi‐group CFA for two‐factor model of patients split by median age (8–14 vs. 15–17).

Model	CFI	RMSEA	SRMR	Delta CFI
Configural	0.827	0.164	0.301	—
Metric	0.831	0.154	0.304	−0.004
Scalar	0.820	0.153	0.305	0.011

*Note*. The median of patients' age was 15. *n* = 98 patients were aged 8–14 and *n* = 107 patients were aged 15–17. Robust values were computed using Satorra–Bentler correction.

Abbreviations: CFI, Comparative Fit Index; Delta CFI, difference between Comparative Fit Index of each model with the model with less restrictions (i.e., metric vs. configural and scalar vs. metric); RMSEA, Root Mean Square Error of Approximation; SRMR, Standardized Root Mean Square Residual.

To ensure the applicability of the two‐factor model to caregivers and HCPs, a second multi‐group CFA was conducted. Results of the model comparisons are summarized in Table 6. The metric model is acceptable, with a difference in CFI of 0.01 compared to the configural model. This suggests that the factor structure is consistent across groups. However, the difference in CFI between the scalar and metric model is 0.06, indicating that intercepts cannot be considered equal between groups. While the model structure fits the groups, mean comparisons between groups are not appropriate.

**Table 6:** Multi‐group CFA for two‐factor model of four groups (patients, caregivers, HCP regarding patients, HCP regarding caregivers).

Model	CFI	RMSEA	SRMR	Delta CFI
Configural	0.733	0.223	0.342	—
Metric	0.723	0.212	0.347	0.010
Scalar	0.661	0.220	0.353	0.062

*Note:* Robust values were computed using Satorra‐Bentler correction.

Abbreviations: CFI, Comparative Fit Index; Delta CFI, difference between Comparative Fit Index of each model with the model with less restrictions (i.e., metric vs. configural and scalar vs. metric); RMSEA, Root Mean Square Error of Approximation; SRMR, Standardized Root Mean Square Residual.

#### 3.2.3 Convergent Validity

##### 3.2.3.1 Patients

Regarding data concerning patients (reports by patients, reports made by HCPs regarding patients), all associations within raters across constructs were significant. Correlations of HAQ‐P scores between the two subscales and the global item ranged from moderate (*τ* = 0.31) to large (*τ* = 0.54) within the patient data and moderate (*τ* = 0.48) to large (*τ* = 0.66) within the HCP ratings regarding patients. Correlations between patient and HCP ratings ranged from *τ* = 0.08 (relationship rating from both parties) to *τ* = 0.25 (global rating from both parties). Notably, only correlations between the HCP global item and patient satisfaction (*τ* = 0.24) and the global patient rating (*τ* = 0.25) were significant. See Table 7 for all correlation coefficients.

**Table 7:** Kendall's *τ* correlations between HAQ‐P scores of patients and HAQ‐P scores of HCP regarding patients.

Variable		HAQ‐P patients			HAQ‐P HCP		
		*R*	OS	Global	*R*	OS	Global
HAQ‐P patients	Relationship	1					
	Outcome satisfaction	0.49*	1				
	Global item	0.31*	0.54*	1			
HAQ‐P HCP	Relationship	0.08	0.10	0.13	1		
	Outcome satisfaction	0.14	0.18	0.18	0.66*	1	
	Global item	0.15	0.24*	0.25*	0.48*	0.65*	1

Abbreviations: OS, outcome satisfaction; R, relationship.

* Significant at *p* ≤ 0.05 after Bonferroni–Holm correction.

Correlational data between patient HAQ‐P scores and pain characteristics (intensity, emotional distress, and functional disability) indicate that both the *outcome satisfaction* subscale and the global item correlate negatively and significantly with all pain characteristics (ranging from *τ* = −0.24 to *τ* = −0.36). That is, higher satisfaction and more positive ratings of change were associated with less pain. While associations between the *relationship* subscale and pain characteristics were also negative (ranging from *τ* = −0.10 to *τ* = −0.16), they did not reach significance. Both patient HAQ‐P scores and the global item correlated significantly with treatment satisfaction and pain self‐efficacy, ranging from *τ* = 0.25 (*relationship* subscale and pain self‐efficacy) to *τ* = 0.52 (*outcome satisfaction* subscale and treatment satisfaction). See Table 8 for all correlation coefficients.

**Table 8:** Kendall's *τ* correlations between HAQ‐P scores of patients and pain characteristics as well as treatment satisfaction and self‐efficacy.

Variable		Pain severity			Treatment satisfaction	Self‐efficacy
		Intensity	Distress	Disability		
HAQ‐P patients	Relationship	−0.10	−0.15	−0.16	0.42*	0.25*
	Outcome satisfaction	−0.24*	−0.33*	−0.35*	0.52*	0.50*
	Global item	−0.29*	−0.32*	−0.36*	0.51*	0.44*

* Significant at *p* ≤ 0.05 after Bonferroni–Holm correction.

##### 3.2.3.2 Caregivers

Similar to the patient subsample, correlations within caregiver data indicated stronger associations within raters across constructs than between raters within the same construct. Correlations between caregiver HAQ‐CG subscales and the global item were strong, ranging from *τ* = 0.54 to *τ* = 0.65. Correlations of HCP ratings were moderate (*τ* = 0.45) to large (*τ* = 0.64). However, none of the associations between caregiver and HCP ratings were significant, with small correlations ranging from *τ* = 0.13 to *τ* = 0.21. See Table 9 for all correlational data.

**Table 9:** Kendall's *τ* correlations between HAQ‐CG scores of caregivers and HAQ‐CG scores of HCP regarding caregivers.

Variable		HAQ‐CG caregivers			HAQ‐CG HCP		
		*R*	OS	Global	*R*	OS	Global
HAQ‐CG caregivers	Relationship	1					
	Outcome satisfaction	0.65*	1				
	Global item	0.54*	0.55*	1			
HAQ‐CG HCP	Relationship	0.19	0.21	0.19	1		
	Outcome satisfaction	0.15	0.19	0.20	0.64*	1	
	Global item	0.13	0.21	0.19	0.45*	0.60*	1

Abbreviations: OS, outcome satisfaction; R, relationship.

* Significant at *p* ≤ 0.05 after Bonferroni‐Holm correction.

Regarding HAQ‐CG scores of caregivers and their association with the other parental measures in this study, there appeared to be little to no association between pain‐related parental behavior (discouraging, distracting, or solicitous behavior) and the HAQ‐CG. Although none of the correlations were significant, three of them were small: the correlation between parental satisfaction and discouraging behavior was *τ* = −0.10; distracting behavior was positively associated with *relationship* (*τ* = 0.12) and *outcome satisfaction* (*τ* = 0.16). However, all HAQ‐CG scores were significantly and positively associated with treatment satisfaction (moderate associations ranging from *τ* = 0.43 to *τ* = 0.48) and their confidence in handling pain (small to moderate associations ranging from *τ* = 0.26 to *τ* = 0.35). See Table 10 for all correlation coefficients.

**Table 10:** Kendall's *τ* correlations between HAQ‐CG scores of caregivers and ISEV‐E, outcome satisfaction and handling pain.

Variable		ISEV‐E			Treatment satisfaction	Confidence in handling pain
		Discouraging	Solicitous	Distracting		
HAQ‐CG caregivers	Relationship	−0.06	0.05	0.12	0.43*	0.26*
	Outcome satisfaction	−0.10	0.04	0.16	0.46*	0.32*
	Global item	−0.01	0.07	0.06	0.48*	0.35*

* Significant at *p* ≤ 0.05 after Bonferroni–Holm correction.

## 4 Discussion

This study successfully developed a German version of the Helping Alliance Questionnaire for pediatric patients (HAQ‐P) and caregivers (HAQ‐CG). Regarding the patient population, results indicated a good fit for the two‐factor structure consisting of the *relationship* and *outcome satisfaction* subscales. Multi‐group analysis revealed comparable two‐factor structures across all subgroups, though it did not allow for group mean comparisons. Convergent validity was confirmed through correlations between HAQ‐P and HAQ‐CG scores and similar constructs. In line with the hypotheses, patient HAQ‐P scores correlated significantly with pain self‐efficacy and treatment satisfaction, while HAQ‐CG scores of caregivers were associated with their confidence in handling their child's pain as well as with their overall satisfaction with treatment. Contrary to the hypotheses, however, only patient *outcome satisfaction* was significantly associated with pain intensity, and caregiver HAQ‐CG scores did not correlate significantly with pain‐related parental behavior.

### 4.1 Item and Scale Properties

Examination of item properties revealed that patients made use of the full response range of all HAQ‐P items, without a ceiling effect. Caregivers and HCPs also utilized most of the score range, with caregivers favoring generally higher values. Internal consistency was excellent for the *relationship* subscale and good to excellent for the *outcome satisfaction* subscale across all four subgroups, affirming the HAQ‐P and HAQ‐CG as reliable measures.

### 4.2 Construct Validity

#### 4.2.1 Confirmatory Factor Analysis

Factor analyses demonstrated that the original two‐factor structure proposed by Bassler and Nübling (2015), encompassing the *relationship* and *outcome satisfaction* subscales, exhibited a good model fit with a CFI of 0.93. Notably, the factor loading of item 11 in the patient sample appeared lower than that of other items. While some fit indices slightly improved when excluding this item (e.g., CFI increased from 0.93 to 0.95), several factors discourage its removal from the questionnaire. One factor is that the statistical comparison between the models with and without item 11 favored its inclusion, suggesting better fit with the data. Furthermore, the factor loading of item 11 was less distinct in the caregiver and HCP data. Retaining item 11 offers the benefit of better comparability with HAQ versions in other contexts (Bassler and Nübling 2015; Bassler et al. 1995) and languages (Kermarrec et al. 2006). While maintaining item 11 seems favorable based on current evidence, future studies may warrant further investigation.

#### 4.2.2 Multi‐Group CFA

The comparison of patient age groups revealed that the validity of the HAQ‐P is independent of the patient's age, both regarding its factor structure and the intercept. This indicates that there is no age‐related difference in the patients’ ratings of relationship and outcome satisfaction. It hence may also indicate that there is no age‐related difference in understanding of the items, supporting the notion that adjusting the items following the cognitive interviews did indeed ease their comprehensibility.

The multi‐group factor analysis across target groups indicated that the factor loadings were consistent across all groups, suggesting that the two‐factor model fits the data well for all four sets of items. Yet, the intercepts of the groups were not comparable, which implies that mean differences observed between groups may be systematic. In this sample, caregiver HAQ‐CG scores were descriptively higher than those of patients and HCPs. This difference might reflect a social desirability bias (Bornstein et al. 2015), as studies show that societal expectations can influence evaluations of problematic behavior among parents (Runge and Soellner 2022). Another possible explanation is the difference in the amount of contact patients and caregivers have with healthcare professionals during IIPT. Patients typically have more direct interaction with HCPs than caregivers do, making it more difficult for caregivers to evaluate the helping alliance. This aspect could be examined further in future studies.

Even though the multi‐group CFA revealed that mean group differences cannot be interpreted, HAQ‐P and HAQ‐CG scores may still be used for within‐group comparisons, such as measuring the development of perceived therapeutic alliance over time. Between groups, scores can be used for inferential statistical calculations that do not rely on group means, such as correlations.

#### 4.2.3 Convergent Validity

The association between patient and HCP ratings of the patient‐HCP alliance was smaller than expected, with only two small, significant correlations. Similarly, caregiver‐HCP alliance ratings by caregivers and HCP showed small, non‐significant correlations. Generally, associations within raters across constructs were stronger than associations across raters, consistent with the multi‐trait‐multi‐method approach (Campbell and Fiske 1959). The results of this study suggest that patients and HCPs may perceive the therapeutic alliance differently. One possible explanation is that the different sets of items measured different relationships. Although the wording of the items is similar across perspectives (e.g., ‘I believe we have similar ideas about the nature of my problems.’ and ‘I believe that my patient/the caregivers and I have similar ideas about their problems.’), the subjects of these questions differ. HCPs rated the items specifically regarding either the patient or the caregiver, whereas patients and caregivers were instructed to consider the entire healthcare team.

Another explanation could be different priorities in treatment outcomes between HCPs, patients, and caregivers. Patients and caregivers may have focused mainly on immediate pain reduction, while HCPs have a broader perspective, also emphasizing longer‐term outcomes like increasing pain self‐efficacy or reducing stress, pain, and anxiety (Dobe and Zernikow 2019).

Additionally, the strong association between the therapeutic relationship and treatment outcomes commonly found in clinical research (Burns et al. 2015; Lambert and Barley 2001; Zuroff and Blatt 2006) was not evident in this study. Although all correlations between patient HAQ‐P scores and pain intensity were negative, only the subscale *outcome satisfaction* correlated significantly with pain characteristics. This means that patients who experienced less pain at discharge were more satisfied with the treatment outcome. Reduction of pain intensity is an important treatment goal of IIPT (Dobe and Zernikow 2019). It is, therefore, understandable that *outcome satisfaction* correlated more strongly with pain characteristics than with *relationship*. Recovering from chronic pain conditions can be a lengthy process. A systematic review by Claus et al. (2022) revealed that a reduction in pain intensity is more noticeable at a follow‐up of at least 1–3 months rather than immediately post‐treatment. Therefore, pain intensity measured in this study might not yet have reached the target level that might be visible in longer‐term data. Additionally, this study could not feasibly include comparisons between outcomes pre‐ and post‐treatment, not allowing for any statements on clinical change. Future studies should explore the role of relationships between patients/caregivers and HCPs more thoroughly by examining all perspectives on the construct and its longer‐term effects.

Pain‐related self‐efficacy reflects the patient's confidence in managing their pain (Stahlschmidt et al. 2023; Stahlschmidt et al. 2019). In line with the hypotheses, there was a significant positive association between pain self‐efficacy and HAQ‐P scores. Patients who feel confident in managing their pain rate their alliance with the HCPs more positively. The association between pain self‐efficacy and *outcome satisfaction* was particularly high, with *τ* = 0.50. The theory of behavioral change suggests that clinical change is highly dependent on pain self‐efficacy (Bandura 1977). Several studies indicate that pain self‐efficacy is indeed an important factor for clinical outcomes in pain treatment, both in adults (Hayward and Stynes 2021; Karasawa et al. 2019) and children (Connelly et al. 2019; Stahlschmidt et al. 2019). Consequently, improving pain self‐efficacy is a primary goal of chronic pain treatment (Dobe and Zernikow 2019; Dogan et al. 2021). The findings of the present study align with these proposed treatment aims and mechanisms. It is also possible that feeling a strong helping alliance with the HCPs helps patients feel more self‐efficacious. Future clinical research would benefit from examining the mechanisms underlying the association between pain self‐efficacy and helping alliance found in this study using longitudinal data.

The caregiver's confidence in handling their child's pain is similar to the construct of pain self‐efficacy but focuses on the caregiver's perspective. Results from the present study indicate that higher confidence in handling the child's pain correlates with a closer relationship with the HCPs and greater satisfaction with the treatment outcome. One of the goals of IIPT for caregivers of patients with chronic pain is guiding them in how they respond to their child's pain (Dobe and Zernikow 2019). Overly solicitous or discouraging behaviors are considered unhelpful; instead, the aim is to encourage active coping behaviors, such as distraction (Hermann et al. 2008). Interestingly, HAQ‐CG scores in the present study were significantly associated with confidence in handling pain but not with self‐reported actual pain management. This is unexpected, as caregiver behavior significantly impacts their child's pain condition (Ngo et al. 2024; Runge and Soellner 2022; Simons et al. 2008), which would logically be related to the caregiver's satisfaction with the outcome. However, the data in this study were collected at discharge from an inpatient stay with IIPT. During this period, patients lived in the hospital for approximately 3 weeks. Due to the COVID‐19 pandemic, patients could rarely go home on weekends, a practice that is normally part of the treatment to ease the transition from inpatient to home setting. Thus, the overall time spent together between patients and caregivers was short, yielding limited opportunities for caregivers to apply new or modified behaviors. Looking at data collected several weeks or months after discharge—giving caregivers more time to regularly apply new behaviors—may provide clearer insights.

Both patients and caregivers showed moderate to large correlations between their HAQ‐P and HAQ‐CG scores and treatment satisfaction, indicating that higher scores on the helping alliance were associated with greater satisfaction. Contrary to a study by Stahlschmidt et al. (2018), which suggested treatment satisfaction may be an independent outcome measure due to low correlations with treatment outcomes, our findings suggest otherwise; satisfaction with treatment seems to be associated with the helping alliance. Implementing the HAQ‐P and HAQ‐CG offers valuable insights into various outcomes in pediatric pain treatment.

### 4.3 Strengths and Limitations

The present study is the first to develop and validate a pediatric version of the HAQ in German. It provides a new tool for measuring patient‐HCP and caregiver‐HCP alliances in pediatric care. However, there are some limitations to consider. One limitation is that data were only collected once, at discharge from the inpatient setting, so changes over time were not tracked. In a sample of adult patients with chronic pain, Burns et al. (2015) found that the therapeutic alliance during treatment is related to pain intensity post‐treatment, where a stronger perceived alliance was associated with less pain. This predictive effect, however, can only be explored in longitudinal studies.

Another limitation is that each patient and caregiver filled in the questionnaire once, but HCPs completed the questionnaire for several of their patients. Filling out the questionnaire repeatedly bears the bias of data being more similar within each HCP than between HCPs. However, this adds to the external validity of the questionnaire, as it reflects how the HAQ may be used in the clinical/research setting. Finally, the present study focused only on patients affected by chronic pain, excluding psychological issues that often accompany chronic pain, such as anxiety and depression. While this creates a strong foundation for future HAQ‐P and HAQ‐CG use in chronic pain treatment, it is desirable to validate the instrument with other conditions.

One strength of this study is its sample size. Of the 216 eligible patients, 205 completed the questionnaire—a high participation rate of 95%. Considering that all new admissions were screened for eligibility, these data are representative of patients with chronic pain aged 8–17 undergoing inpatient treatment. Another notable strength is the use of cognitive interviews during questionnaire development. It is crucial for validity that the psychological measures are comprehensible to the target audience (Crombez et al. 2023; Willis 2005). Adjustments made as a result of these cognitive interviews ensured that both patients and caregivers understood all items.

### 4.4 Practical Implications

The pediatric version of the HAQ offers clinicians and researchers in pediatric healthcare a valuable new tool to monitor both patients’ and their caregivers’ alliance towards them. Clinicians may use the results to improve treatment and tailor it to the family's needs. Implementing the HAQ‐P and HAQ‐CG in clinical research studies may improve outcome predictability and provide new insights. Moreover, validating the two‐factor structure in a multi‐group setting could enable dyadic analyses of therapeutic alliances.

## 5 Conclusion

The pediatric German version of the HAQ is a reliable and valid instrument for assessing the helping alliance between pediatric patients with chronic pain and their HCPs, as well as between their caregivers and the HCPs. This study confirms the previously found two‐factor structure with the *relationship* and *outcome satisfaction* subscales across all subgroups, though group means cannot be directly compared. Further research could validate the HAQ‐P and HAQ‐CG in longitudinal studies and in samples with broader clinical impact.

## Ethics Statement

The study was approved by the ethics committee of Children's and Adolescents' Hospital Datteln, Germany (no. 2021/07/15/JW).

## Conflicts of Interest

The authors declare no conflicts of interest.

## Supporting information


**Table S1:** Items of the HAQ‐P (validated German version and non‐validated English version) measuring the patient‐HCP alliance.
**Table S2:** Items of the HAQ‐CG (validated German version and non‐validated English version) measuring the caregiver‐HCP alliance.
**Table S3:** Scale and item properties for the two‐factor model in the patient sample (n = 205).
**Table S4:** Scale and item properties for the two‐factor model in the caregiver sample (n = 191).
**Table S5:** Scale and item properties for the two‐factor model in the HCP sample (HCP‐P; n = 197).
**Table S6:** Scale and item properties for the two‐factor model of the HC sample (HCP‐CG; n = 197).

## Data Availability

Data and syntaxes are available upon request from the corresponding author. The data are not publicly available due to privacy or ethical restrictions.

## References

[jclp70152-bib-0001] Accurso, E. C. , K. M. Hawley , and A. F. Garland . 2013. “Psychometric properties of the Therapeutic Alliance Scale for Caregivers and Parents.” Psychological Assessment 25, no. 1: 244–252. 10.1037/a0030551. 23088205 PMC3647370

[jclp70152-bib-0002] Alp, R. , S. I. Alp , Y. Palanci , et al. 2010. “Use of the International Classification of Headache Disorders, Second Edition, Criteria in the Diagnosis of Primary Headache in Schoolchildren: Epidemiology Study From Eastern Turkey.” Cephalalgia 30, no. 7: 868–877. 10.1177/0333102409355837. 20647179

[jclp70152-bib-0003] Bandura, A. 1977. “Self‐Efficacy: Toward a Unifying Theory of Behavioral Change.” Psychological Review 84, no. 2: 191–215. 10.1037//0033-295x.84.2.191. 847061

[jclp70152-bib-0004] Bassler, M. , and R. Nübling (2015). Helping Alliance Questionnaire.

[jclp70152-bib-0005] Bassler, M. , B. Potratz , and H. Krauthauser . 1995. “Der ‘Helping Alliance Questionnaire’ (HAQ) von Luborsky.” Psychotherapeut 40: 23–32.

[jclp70152-bib-0006] Bornstein, M. H. , D. L. Putnick , J. E. Lansford , et al. 2015. “Mother and Father Socially Desirable Responding in Nine Countries: Two Kinds of Agreement and Relations to Parenting Self‐Reports.” International Journal of Psychology 50, no. 3: 174–185. 10.1002/ijop.12084. 25043708 PMC4297254

[jclp70152-bib-0007] Brown, T. A. 2015. Confirmatory Factor Analysis for Applied Research. Methodology in the Social Sciences. The Guilford Press.

[jclp70152-bib-0008] Burns, J. W. , W. R. Nielson , M. P. Jensen , A. Heapy , R. Czlapinski , and R. D. Kerns . 2015. “Specific and General Therapeutic Mechanisms in Cognitive Behavioral Treatment of Chronic Pain.” Journal of Consulting and Clinical Psychology 83, no. 1: 1–11. 10.1037/a0037208. 24979313

[jclp70152-bib-0009] Campbell, D. T. , and D. W. Fiske . 1959. “Convergent and Discriminant Validation by the Multitrait‐Multimethod Matrix.” Psychological Bulletin 56, no. 2: 81–105. 10.1037/h0046016. 13634291

[jclp70152-bib-0010] Claus, B. B. , L. Stahlschmidt , E. Dunford , et al. 2022. “Intensive Interdisciplinary Pain Treatment for Children and Adolescents With Chronic Noncancer Pain: A Preregistered Systematic Review and Individual Patient Data Meta‐Analysis.” Pain 163, no. 12: 2281–2301. 10.1097/j.pain.0000000000002636. 35297804

[jclp70152-bib-0011] Cohen, J. 1988. Statistical Power Analysis for the Behavioral Sciences. L. Erlbaum Associates.

[jclp70152-bib-0012] Connelly, M. , L. E. Schanberg , S. Ardoin , et al. 2019. “Multisite Randomized Clinical Trial Evaluating an Online Self‐Management Program for Adolescents With Juvenile Idiopathic Arthritis.” Journal of Pediatric Psychology 44, no. 3: 363–374. 10.1093/jpepsy/jsy066. 30204919 PMC6415659

[jclp70152-bib-0013] Crombez, G. , E. Veirman , D. van Ryckeghem , W. Scott , and A. De Paepe . 2023. “The Effect of Psychological Factors on Pain Outcomes: Lessons Learned for the Next Generation of Research.” PAIN Reports 8, no. 6: e1112. 10.1097/PR9.0000000000001112. 38027466 PMC10631620

[jclp70152-bib-0014] Dobe, M. , and B. Zernikow . 2019. Therapie von Schmerzstörungen im Kindes‐ und Jugendalter: Ein Manual für Psychotherapeuten, Ärzte und Pflegepersonal. Springer Berlin Heidelberg.

[jclp70152-bib-0015] Dogan, M. , G. Hirschfeld , M. Blankenburg , et al. 2021. “Effectiveness of a Psychosocial Aftercare Program for Youth Aged 8 to 17 Years With Severe Chronic Pain: A Randomized Clinical Trial.” JAMA Network Open 4, no. 9: e2127024. 10.1001/jamanetworkopen.2021.27024. 34570203 PMC8477265

[jclp70152-bib-0016] Flückiger, C. , A. O. Horvath , A. C. Del Re , D. Symonds , and C. Holzer . 2015. “Bedeutung der Arbeitsallianz in der Psychotherapie.” Psychotherapeut 60, no. 3: 187–192. 10.1007/s00278-015-0020-0.

[jclp70152-bib-0017] Gaston, L. 1991. “Reliability and Criterion‐Related Validity of the California Psychotherapy Alliance Scales—Patient Version.” Psychological Assessment: A Journal of Consulting and Clinical Psychology 3, no. 1: 68–74. 10.1037/1040-3590.3.1.68.

[jclp70152-bib-0018] Gliem, J. A. , and R. R. Gliem . 2003. “Calculating, Interpreting, and Reporting Cronbach's Alpha Reliability Coefficient for Likert‐Type Scales.” In Proceedings of the Midwest Research‐to‐Practice Conference in Adult, Continuing, and Community Education, 8–10. Ohio State University. https://scholarworks.indianapolis.iu.edu/items/63734e75-1604-45b6-aed8-40dddd7036ee.

[jclp70152-bib-0019] Goretzko, D. , K. Siemund , and P. Sterner . 2024. “Evaluating Model Fit of Measurement Models in Confirmatory Factor Analysis.” Educational and Psychological Measurement 84, no. 1: 123–144. 10.1177/00131644231163813. 38250508 PMC10795573

[jclp70152-bib-0020] Grothus, S. , A. Sommer , L. Stahlschmidt , et al. 2024. “Pediatric Chronic Pain Grading—A Revised Classification of the Severity of Pediatric Chronic Pain.” Pain 165, no. 9: 2087–2097. 10.1097/j.pain.0000000000003226. 38595202

[jclp70152-bib-0021] Harvey, A. M. 1995. “Classification of Chronic Pain—Descriptions of Chronic Pain Syndromes and Definitions of Pain Terms.” Clinical Journal of Pain 11, no. 2: 163. 10.1097/00002508-199506000-00024.

[jclp70152-bib-0022] Hayward, R. , and S. Stynes . 2021. “Self‐Efficacy as a Prognostic Factor and Treatment Moderator in Chronic Musculoskeletal Pain Patients Attending Pain Management Programmes: A Systematic Review.” Musculoskeletal Care 19, no. 3: 278–292. 10.1002/msc.1533. 33378591

[jclp70152-bib-0023] Hermann, C. , K. Zohsel , J. Hohmeister , and H. Flor . 2008. “Dimensions of Pain‐Related Parent Behavior: Development and Psychometric Evaluation of a New Measure for Children and Their Parents.” Pain 137, no. 3: 689–699. 10.1016/j.pain.2008.03.031. 18534757

[jclp70152-bib-0024] Hirschfeld, G. , and R. von Brachel . 2019. “Improving Multiple‐Group Confirmatory Factor Analysis in R—A Tutorial in Measurement Invariance With Continuous And Ordinal Indicators.” Practical Assessment, Research, and Evaluation 19, no. 1: 7. 10.7275/QAZY-2946.

[jclp70152-bib-0025] Holm, S. 1979. “A Simple Sequentially Rejective Multiple Test Procedure.” Scandinavian Journal of Statistics 6, no. 2: 65–70. https://www.jstor.org/stable/4615733.

[jclp70152-bib-0026] Hu, L. , and P. M. Bentler . 1999. “Cutoff Criteria for Fit Indexes in Covariance Structure Analysis: Conventional Criteria Versus New Alternatives.” Structural Equation Modeling: A Multidisciplinary Journal 6, no. 1: 1–55. 10.1080/10705519909540118.

[jclp70152-bib-0027] Karasawa, Y. , K. Yamada , M. Iseki , et al. 2019. “Association Between Change in Self‐efficacy and Reduction in Disability Among Patients With Chronic Pain.” PLoS One 14, no. 4: e0215404. 10.1371/journal.pone.0215404. 30990842 PMC6467389

[jclp70152-bib-0028] Kermarrec, S. , B. Kabuth , C. Bursztejn , and F. Guillemin . 2006. “French Adaptation and Validation of the Helping Alliance Questionnaires for Child, Parents, and Therapist.” Canadian Journal of Psychiatry 51, no. 14: 913–922. 10.1177/070674370605101407. 17249634

[jclp70152-bib-0029] Kline, R. B. 2023. Principles and Practice of Structural Equation Modeling. Guilford Publications.

[jclp70152-bib-0030] Könning, A. , N. Rosenthal , D. Brown , L. Stahlschmidt , and J. Wager . 2021. “Severity of Chronic Pain in German Adolescent School Students: A Cross‐Sectional Study.” Clinical Journal of Pain 37, no. 2: 118–125. 10.1097/AJP.0000000000000898. 33165023

[jclp70152-bib-0031] Kyriazos, T. A. 2018. “Applied Psychometrics: Sample Size and Sample Power Considerations In Factor Analysis (EFA, CFA) and SEM in General.” Psychology 09, no. 08: 2207–2230. 10.4236/psych.2018.98126.

[jclp70152-bib-0032] Lambert, M. J. , and D. E. Barley . 2001. “Research Summary on the Therapeutic Relationship and Psychotherapy Outcome.” Psychotherapy: Theory, Research, Practice, Training 38, no. 4: 357–361. 10.1037//0033-3204.38.4.357.

[jclp70152-bib-0033] Luborsky, L. 1984. Principles of Psychoanalytic Psychotherapy: A Manual for Supportive‐Expressive Psychotherapy. Basic Books.

[jclp70152-bib-0034] Lutz, W. , J. Rubel , A. ‑K. Schiefele , D. Zimmermann , J. R. Böhnke , and W. W. Wittmann . 2015. “Feedback and Therapist Effects in the Context of Treatment Outcome and Treatment Length.” In Patient‐Focused and Feedback Research in Psychotherapy, 33–46. Routledge. 10.4324/9781315515656-6. 26218788

[jclp70152-bib-0035] Meyer, B. , J. Bierbrodt , J. Schröder , et al. 2015. “Effects of an Internet intervention (Deprexis) on Severe Depression Symptoms: Randomized Controlled Trial.” Internet Interventions 2, no. 1: 48–59. 10.1016/j.invent.2014.12.003.

[jclp70152-bib-0036] Michel, K. , P. Dey , K. Stadler , and L. Valach . 2004. “Therapist Sensitivity Towards Emotional Life‐career Issues and the Working Alliance With Suicide Attempters.” Archives of Suicide Research 8, no. 3: 203–213. 10.1080/13811110490436792. 16081387

[jclp70152-bib-0037] Ngo, D. , G. M. Palmer , A. Gorrie , T. Kenmuir , M. Crawford , and T. Jaaniste . 2024. “Caregiver Burden Associated With Pediatric Chronic Pain: A Retrospective Study Using the Pediatric Electronic Persistent Pain Outcomes Collaboration Database.” Clinical Journal of Pain 40, no. 3: 137–149. 10.1097/AJP.0000000000001189. 38149451

[jclp70152-bib-0038] Nübling, R. , M. Kraft , J. Henn , et al. 2017. “Psychometrische Überprüfung des Helping Alliance Questionnaire (HAQ) in unterschiedlichen Versorgungssettings.” Psychotherapie, Psychosomatik, Medizinische Psychologie 67, no. 11: 465–476. 10.1055/s-0043-111083. 28854445

[jclp70152-bib-0039] Posit team . (2024). RStudio: Integrated Development Environment for R [Computer software]. Posit Software, PBC. http://www.posit.co/.

[jclp70152-bib-0040] Puschner, B. , S. Bauer , L. M. Horowitz , and H. Kordy . 2005. “The Relationship Between Interpersonal Problems and the Helping Alliance.” Journal of Clinical Psychology 61, no. 4: 415–429. 10.1002/jclp.20050. 15503317

[jclp70152-bib-0041] R Core Team . (2024). *R* (Version 4.4.0) [Computer Software]. R Foundation for Statistical Computing. https://www.R-project.org/.

[jclp70152-bib-0042] Rau, L. ‑M. , B. Korwisi , A. Barke , et al. 2025. “International Classification of Diseases‐11 Chronic Pain Severity Specifiers for Children and Adolescents: A Validation Study.” Pain 166, no. 9: 2034–2043. 10.1097/j.pain.0000000000003584. 40112198

[jclp70152-bib-0043] Rosseel, Y. 2012. “Lavaan: An R package for Structural Equation Modeling.” Journal of Statistical Software 48, no. 2: 1–36. 10.18637/jss.v048.i02.

[jclp70152-bib-0044] Runge, R. A. , and R. Soellner . 2022. “Cultural Bias in Parent Reports: The Role of Socialization Goals When Parents Report on Their Child's Problem Behavior.” Child Psychiatry & Human Development 55, no. 4: 1020–1030. 10.1007/s10578-022-01464-y. 36371526 PMC11245439

[jclp70152-bib-0045] Satorra, A. , and P. M. Bentler . 2010. “Ensuring Positiveness of the Scaled Difference Chi‐Square Test Statistic.” Psychometrika 75, no. 2: 243–248. 10.1007/s11336-009-9135-y. 20640194 PMC2905175

[jclp70152-bib-0046] Schroeder, S. , T. Hechler , H. Denecke , et al. 2010. “Deutscher Schmerzfragebogen für Kinder, Jugendliche und deren Eltern (DSF‐KJ). Entwicklung und Anwendung eines multimodalen Fragebogens zur Diagnostik und Therapie chronischer Schmerzen im Kindes‐ und Jugendalter [German Pain Questionnaire for Children, Adolescents and Parents (DSF‐KJ). A multimodal questionnaire for diagnosis and treatment of children and adolescents suffering from chronic pain].” Der Schmerz 24, no. 1: 23–37. 10.1007/s00482-009-0864-8. 20108103

[jclp70152-bib-0047] Simons, L. E. , R. L. Claar , and D. L. Logan . 2008. “Chronic Pain in Adolescence: Parental Responses, Adolescent Coping, and Their Impact on Adolescent's Pain Behaviors.” Journal of Pediatric Psychology 33, no. 8: 894–904. 10.1093/jpepsy/jsn029. 18375447 PMC2493514

[jclp70152-bib-0048] Stahlschmidt, L. , M. Dogan , B. Hübner‐Möhler , et al. 2023. “Development and Validation of the Scale for Pain Self‐Efficacy (SPaSE) in German and English languages for Children and Adolescents.” Journal of Pain 24, no. 6: 1069–1079. 10.1016/j.jpain.2023.01.007. 36646401

[jclp70152-bib-0049] Stahlschmidt, L. , B. Hübner‐Möhler , M. Dogan , and J. Wager . 2019. “Pain Self‐Efficacy Measures for Children and Adolescents: A Systematic Review.” Journal of Pediatric Psychology 44, no. 5: 530–541. 10.1093/jpepsy/jsz002. 30802913

[jclp70152-bib-0050] Stahlschmidt, L. , B. Zernikow , and J. Wager . 2016. “Specialized Rehabilitation Programs for Children and Adolescents With Severe Disabling Chronic Pain: Indications, Treatment and Outcomes.” Children (Basel, Switzerland) 3, no. 4: 33. 10.3390/children3040033. 27879631 PMC5184808

[jclp70152-bib-0051] Stahlschmidt, L. , B. Zernikow , and J. Wager . 2018. “Satisfaction With an Intensive Interdisciplinary Pain Treatment For Children and Adolescents: An Independent Outcome Measure.” Clinical Journal of Pain 34, no. 9: 795–803. 10.1097/AJP.0000000000000600. 29485533

[jclp70152-bib-0052] H. H. M. Burgmeier‐Lohse , and H. Wille . 1992. “Deutschsprachige Version der Vanderbilt‐Psychotherapie‐Skalen: Beschreibung und Anwendung in zwei Kurztherapien [German versions of the Vanderbilt Psychotherapy Scales: Description and application in 2 brief therapies].” Zeitschrift Für Klinische Psychologie, Psychopathologie Und Psychotherapie 40, no. 4: 411–430. 1441689

[jclp70152-bib-0053] Thornton, G. C. D. , M. J. Goldacre , R. Goldacre , and L. J. Howarth . 2016. “Diagnostic Outcomes Following Childhood Non‐Specific Abdominal Pain: A Record‐Linkage Study.” Archives of Disease in Childhood 101, no. 4: 305–309. 10.1136/archdischild-2015-308198. 26220924

[jclp70152-bib-0054] Treede, R. D. , W. Rief , A. Barke , et al. 2019. “Chronic Pain as a Symptom or a Disease: The IASP Classification of Chronic Pain for the International Classification of Diseases (ICD‐11).” Pain 160, no. 1: 19–27. 10.1097/j.pain.0000000000001384. 30586067

[jclp70152-bib-0055] Vandenberg, R. J. , and C. E. Lance . 2000. “A Review and Synthesis of the Measurement Invariance Literature: Suggestions, Practices, and Recommendations for Organizational Research.” Organizational Research Methods 3, no. 1: 4–70. 10.1177/109442810031002.

[jclp70152-bib-0056] Willis, G. B. 2005. Cognitive Interviewing: A Tool for Improving Questionnaire Design. Sage Publications.

[jclp70152-bib-0057] World Health Organization . (2021). International Classification of Diseases, Eleventh Revision (ICD‐11). https://icd.who.int/browse11.

[jclp70152-bib-0058] Zuroff, D. C. , and S. J. Blatt . 2006. “The Therapeutic Relationship in the Brief Treatment of Depression: Contributions to Clinical Improvement and Enhanced Adaptive Capacities.” Journal of Consulting and Clinical Psychology 74, no. 1: 130–140. 10.1037/0022-006x.74.1.130. 16551150

