# Supplementary material for: The Helping Alliance Questionnaire for Children and Their Caregivers—Validation in a German Sample of Pediatric Chronic Pain Patients
Source: J Clin Psychol. 2026 May 7;82(8):1218–30. doi: 10.1002/jclp.70152 (PMC13341042; doi:10.1002/jclp.70152)
Supplement: Supplementary file 1 — Table S1: Items of the HAQ‐P (validated German version and non‐validated English version) measuring the patient‐HCP alliance. Table S2: Items of the HAQ‐CG (validated German version and non‐validated English version) measuring the caregiver‐HCP alliance. Table S3: Scale and item properties for the two‐factor model in the patient sample (n = 205). Table S4: Scale and item properties for the two‐factor model in the caregiver sample (n = 191). Table S5: Scale and item properties for the two‐factor model in the HCP sample (HCP‐P; n = 197). Table S6: Scale and item properties for the two‐factor model of the HC sample (HCP‐CG; n = 197). [file JCLP-82-1218-s001.docx]

**Table S1**

*Items of the HAQ-P (validated German version and non-validated English version) measuring the patient-HCP alliance*

| Item | Patient German (validated) | Patient English | HCP German (validated)  Ich habe den Eindruck, dass… | HCP English  I believe that … | Short phrase |
| --- | --- | --- | --- | --- | --- |
| 1 | Ich glaube, dass meine Behandler mir helfen. | I believe that the HCPs are helping me. | ... ich meinem Patienten / meiner Patienten helfen kann. | … I can help my patient. | Helping HCPs |
| 2 | Ich glaube, dass mir die Schmerzbehandlung hilft. | I believe that the pain treatment is helping me. | ... die Behandlung meinem Patienten / meiner Patientin hilft. | … the treatment is helping my patient. | Treatment helps |
| 3 | Ich habe einige neue Einsichten gewonnen. | I have gained some new insights. | … mein:e Patient:in einige neue Einsichten gewonnen hat. | … my patient has gained some new insights. | New insights |
| 4 | Durch die Schmerzbehandlung fühle ich mich besser. | The pain treatment makes me feel better. | ... sich mein:e Patient:in durch die Behandlung besser fühlt. | … the pain treatment makes my patient feel better. | Feel better |
| 5 | Ich glaube, dass ich meine Probleme bewältigen kann. | I believe that I can overcome my problems. | ... mein:e Patient:in die Probleme bewältigen kann, wegen der er/sie zur Behandlung gekommen ist. | … my patient can overcome the problems for which they have come for treatment. | Overcome problems |
| 6 | Ich kann mich auf die Behandler verlassen. | I feel I can depend upon the HCPs. | ... ich für meinen Patienten / meine Patientin verlässlich bin. | … my patient can depend upon me. | Dependable HCPs |
| 7 | Ich habe das Gefühl, dass mich die Behandler verstehen. | I feel the HCPs understand me. | ... ich meinen Patienten / meine Patientin verstehe. | … I understand my patient. | Understanding HCPs |
| 8 | Ich habe das Gefühl, dass die Behandler möchten, dass ich meine Ziele erreiche. | I feel the HCPs want me to achieve my goals. | ... ich mich dafür einsetze, dass der Patient seine / die Patientin ihre Ziele erreicht. | … I am committed to ensuring that the patient achieves their goals. | Achieve goals |
| 9 | Ich habe das Gefühl, dass die Behandler und ich zusammenarbeiten. | I feel I am working together with the HCPs in a joint effort. | ... mein:e Patient:in und ich an einem Strang ziehen. | … my patient and I are working together in a joint effort. | Joint effort |
| 10 | Ich glaube, dass die Behandler meine Probleme so sehen wie ich. | I believe we have similar ideas about the nature of my problems. | … mein Patient seine / meine Patientin ihre Probleme ähnlich wie ich sieht und beurteilt. | … my patient and I have similar ideas about their problems. | Similar ideas |
| 11 | Ich habe das Gefühl, dass ich mich jetzt selbstständig mit meinen Problemen auseinandersetzen kann. | I feel I can now deal with my problems on my own. | ... mein:e Patient:in sich jetzt versteht und sich selbstständig mit seinen Problemen auseinandersetzen kann (d. h. auch dann, wenn er/sie mit mir keine Gespräche mehr hat). | … my patient can now deal with their problems on their own (even when they don’t have any more sessions with me). | Independence |
| Global item | Wie schätzt du den Erfolg deiner Schmerzbehandlung bisher ein? | How would you rate the success of your pain treatment so far? | Wie schätzen Sie insgesamt die Veränderung der Gesamtproblematik des Kindes seit Beginn der Schmerzbehandlung ein? | How would you rate the overall change in the child's problems since the beginning of pain treatment? | Treatment success |

*Note.* Items were adapted from the adult HAQ of Bassler et al. (1995). Abbreviation: HCPs, healthcare professionals.

**Table S2**

*Items of the HAQ-CG (validated German version and non-validated English version) measuring the caregiver-HCP alliance*

| Item | Caregiver German (validated) | Caregiver English | HCP German (validated)  Ich habe den Eindruck, dass… | HCP English  I believe that … | Short phrase |
| --- | --- | --- | --- | --- | --- |
| 1 | Ich glaube, dass die Behandler mir und meinem Kind helfen. | I believe that the HCPs are helping me and my child. | ... ich den Eltern helfen kann. | … I can help the caregivers. | Helping HCPs |
| 2 | Ich glaube, dass die Schmerzbehandlung auch mir hilft. | I believe that the pain treatment is helping me as well. | ... die Behandlung den Eltern hilft. | … the treatment is helping the caregivers. | Treatment helps |
| 3 | Ich habe einige neue Einsichten gewonnen. | I have gained some new insights. | ... die Eltern einige neue Einsichten gewonnen haben. | … the caregivers have gained some new insights. | New insights |
| 4 | Durch die Schmerzbehandlung meines Kindes fühle ich mich besser. | My child’s pain treatment makes me feel better. | ... sich die Eltern durch die Behandlung besser fühlen. | … the pain treatment makes the caregivers feel better. | Feel better |
| 5 | Ich glaube, dass ich mein Kind unterstützen kann, seine Probleme zu bewältigen. | I believe that I can support my child to overcome their problems. | ... die Eltern die Probleme bewältigen können, wegen der sie zur Behandlung gekommen sind. | … the caregivers can overcome the problems for which they have come for treatment. | Overcome problems |
| 6 | Ich kann mich auf die Behandler verlassen. | I feel I can depend upon the HCPs. | ... ich für die Eltern verlässlich bin. | … the caregivers can depend upon me. | Dependable HCPs |
| 7 | Ich habe das Gefühl, dass mich die Behandler verstehen. | I feel the HCPs understand me. | ... ich die Eltern verstehe. | … I understand the caregivers. | Understanding HCPs |
| 8 | Ich habe das Gefühl, dass die Behandler möchten, dass mein Kind seine Ziele erreicht. | I feel the HCPs want my child to achieve their goals. | ... ich mich dafür einsetze, dass die Eltern die Behandlungsziele ihres Kindes bestmöglich unterstützen. | … I am committed to support the caregivers in helping their child achieve their goals. | Achieve goals |
| 9 | Ich habe das Gefühl, dass die Behandler und ich zusammenarbeiten. | I feel I am working together with the HCPs in a joint effort. | ... die Eltern und ich an einem Strang ziehen. | … the caregivers and I are working together in a joint effort. | Joint effort |
| 10 | Ich glaube, dass die Behandler die Probleme meines Kindes so sehen wie ich. | I believe we have similar ideas about the nature of my child’s problems. | ... die Eltern die Probleme ihres Kindes ähnlich wie ich sehen und beurteilen. | … the caregivers and I have similar ideas about their child’s problems. | Similar ideas |
| 11 | Ich habe das Gefühl, dass ich mein Kind jetzt besser verstehe und mich selbstständig mit den Problemen meines Kindes auseinandersetzen kann. | I believe I understand my child better now and I can deal with my child's problems independently. | ... die Eltern ihr Kind jetzt verstehen und sich selbstständig mit den Problemen ihres Kindes auseinandersetzen können (d. h. auch dann, wenn sie mit mir keine Gespräche mehr haben). | …the caregivers understand their child better now and can deal with the child’s problems independently (even when they don’t have any more sessions with me). | Independence |
| Global item | Wie geht es Ihnen insgesamt im Vergleich zum Beginn der Schmerzbehandlung? | How are you doing overall compared to the beginning of the pain treatment? | Wie schätzen Sie insgesamt die Veränderung der Gesamtproblematik der Eltern seit Beginn der Schmerzbehandlung ein? | How would you rate the overall change in the caregivers’ problems since the beginning of pain treatment? | Treatment success |

*Note.* Items were adapted from the adult HAQ of Bassler et al. (1995). Abbreviation: HCPs, healthcare professionals.

**Table S3**

*Scale and item properties for the two-factor model in the patient sample (n = 205)*

| Subscale (Cronbach’s alpha) | Item mean (MIC) | Item | *M* | *SD* | Skewness | Item-total-correlations | ʎ 2-factor model |
| --- | --- | --- | --- | --- | --- | --- | --- |
| Relationship (.92) | 4.96 (.55) | 1 | 5.11 | 0.98 | -1.59 | .80 | .824 |
|  |  | 6 | 5.08 | 1 | -1.47 | .78 | .800 |
|  |  | 7 | 4.78 | 1.12 | -1.09 | .85 | .840 |
|  |  | 8 | 5.3 | 0.95 | -1.97 | .79 | .796 |
|  |  | 9 | 5.02 | 1.03 | -1.58 | .88 | .882 |
|  |  | 10 | 4.48 | 1.19 | -0.96 | .78 | .782 |
| Satisfaction (.85) | 4.82 (.48) | 2 | 4.95 | 1.01 | -1.36 | .82 | .888 |
|  |  | 3 | 5.08 | 0.97 | -1.45 | .63 | .684 |
|  |  | 4 | 4.7 | 1.13 | -1.04 | .85 | .865 |
|  |  | 5 | 4.72 | 1.05 | -0.85 | .75 | .681 |
|  |  | 11 | 4.64 | 1.09 | -1.02 | .61 | .527 |

Note. MIC was calculated using the Kendall’s *τ* coefficient. Abbreviations: MIC, Mean inter-item correlation (calculated with Kendall’s tau); M, Mean; SD, Standard deviation.

**Table S4**

*Scale and item properties for the two-factor model in the caregiver sample (n = 191)*

| Subscale (Cronbach’s alpha) | Item mean (MIC) | Item | *M* | *SD* | Skewness | Item-total-correlations | ʎ 2-factor model |
| --- | --- | --- | --- | --- | --- | --- | --- |
| Relationship (.91) | 5.17 (.57) | 1 | 5.22 | 0.83 | -1.03 | .80 | .820 |
|  |  | 6 | 5.16 | 0.86 | -1.05 | .89 | .885 |
|  |  | 7 | 5.04 | 0.96 | -1.03 | .81 | .811 |
|  |  | 8 | 5.55 | 0.67 | -1.70 | .70 | .698 |
|  |  | 9 | 5.13 | 0.95 | -1.30 | .87 | .860 |
|  |  | 10 | 4.95 | 0.98 | -1.05 | .74 | .768 |
| Satisfaction (.85) | 4.91 (.46) | 2 | 4.71 | 1.01 | -0.75 | .68 | .737 |
|  |  | 3 | 4.99 | 0.95 | -1.13 | .78 | .772 |
|  |  | 4 | 5.03 | 1.00 | -1.26 | .80 | .825 |
|  |  | 5 | 5.04 | 0.89 | -1.05 | .57 | .551 |
|  |  | 11 | 4.79 | 0.98 | -1.11 | .81 | .779 |

Note. MIC was calculated using the Kendall’s *τ* coefficient. Abbreviations: MIC, Mean inter-item correlation (calculated with Kendall’s tau); M, Mean; SD, Standard deviation.

**Table S5**

*Scale and item properties for the two-factor model in the HCP sample (HCP-P;n = 197)*

| Subscale (Cronbach’s alpha) | Item mean (MIC) | Item | *M* | *SD* | Skewness | Item-total-correlations | ʎ 2-factor model |
| --- | --- | --- | --- | --- | --- | --- | --- |
| Relationship (.90) | 4.90 (.55) | 1 | 4.87 | 0.84 | -0.26 | .81 | .856 |
|  |  | 6 | 5.28 | 0.78 | -0.53 | .78 | .695 |
|  |  | 7 | 4.94 | 0.75 | 0.02 | .67 | .620 |
|  |  | 8 | 5.26 | 0.73 | -0.44 | .78 | .677 |
|  |  | 9 | 4.68 | 0.86 | -0.19 | .81 | .844 |
|  |  | 10 | 4.4 | 0.9 | -0.19 | .78 | .840 |
| Satisfaction (.91) | 4.51 (.60) | 2 | 4.86 | 0.85 | -0.14 | .80 | .854 |
|  |  | 3 | 4.9 | 0.84 | -0.28 | .79 | .841 |
|  |  | 4 | 4.63 | 0.82 | -0.17 | .89 | .873 |
|  |  | 5 | 4.16 | 0.92 | -0.28 | .85 | .798 |
|  |  | 11 | 4.01 | 1.01 | -0.17 | .78 | .732 |

Note. MIC was calculated using the Kendall’s *τ* coefficient. Abbreviations: MIC, Mean inter-item correlation (calculated with Kendall’s tau); M, Mean; SD, Standard deviation.

**Table S6**

*Scale and item properties for the two-factor model of the HC sample (HCP-CG; n = 197)*

| Subscale (Cronbach’s alpha) | Item mean (MIC) | Item | *M* | *SD* | Skewness | Item-total-correlations | ʎ 2-factor model |
| --- | --- | --- | --- | --- | --- | --- | --- |
| Relationship (.90) | 4.58 (.55) | 1 | 4.52 | 0.91 | -0.23 | .77 | .825 |
|  |  | 6 | 5.05 | 0.78 | -0.21 | .73 | .667 |
|  |  | 7 | 4.46 | 0.93 | -0.57 | .77 | .750 |
|  |  | 8 | 4.96 | 0.75 | -0.09 | .73 | .650 |
|  |  | 9 | 4.34 | 0.97 | -0.25 | .85 | .854 |
|  |  | 10 | 4.18 | 1.01 | -0.36 | .79 | .827 |
| Satisfaction (.90) | 4.18 (.57) | 2 | 4.46 | 0.92 | -0.20 | .81 | .865 |
|  |  | 3 | 4.58 | 0.88 | -0.16 | .79 | .840 |
|  |  | 4 | 4.13 | 0.99 | -0.26 | .84 | .836 |
|  |  | 5 | 3.89 | 0.91 | -0.02 | .79 | .712 |
|  |  | 11 | 3.82 | 0.99 | 0.01 | .77 | .707 |

Note. MIC was calculated using the Kendall’s *τ* coefficient. Abbreviations: MIC, Mean inter-item correlation (calculated with Kendall’s tau); M, Mean; SD, Standard deviation.
